# Adherence to Referral Criteria for Burns in the Emergency Department

**Published:** 2008-05-09

**Authors:** Elizabeth Chipp, Jules Walton, David Gorman, Naiem S Moiemen

**Affiliations:** West Midlands Regional Burns Unit, University Hospitals Birmingham Foundation NHS Trust, Raddlebarn Rd, Birmingham, United Kingdom; Emergency Department, University Hospitals Birmingham Foundation NHS Trust, Raddlebarn Rd, Birmingham, United Kingdom

## Abstract

**Objective:** To audit the referral patterns of burns in an emergency department compared with national referral guidelines. **Methods:** A retrospective case note audit of patients attending an emergency department with a diagnosis of “burn” in a 1-year period. **Results:** Only one quarter of the patients were managed according to the suggested national referral criteria for burns. Large and full thickness burns were managed appropriately but those at important anatomical sites and in patients at the extremes of age were managed less well. **Conclusion:** Increased awareness of the national referral guidelines, along with further education of staff within this department, may improve management of burn injuries. It is likely that referral patterns are similar in other emergency departments and may be improved by training staff in the assessment and management of burns. Increased adherence to the guidelines is likely to improve patient outcome at the expense of increased patient numbers and workloads in regional burns units that have implications for funding and service provision.

Burns are potentially devastating injuries that can have long-term physical, psychological, and economic consequences. Mortality rates from major burns are improving and increasingly efforts are focused on improving outcome from relatively minor burns.

It has been traditional to refer burn injuries on the basis of total burn size area (TBSA) alone. It is now recognized that this is overly simplistic and that burns should be referred to a specialist burns service on the basis of injury complexity that may itself be suggested by several factors including patient age, anatomical location, and coexisting medical conditions. Because of the serious but potentially preventable long-tem sequelae that can result from even minor burns, it is important to recognize those injuries that require specialist input and refer these patients to the appropriate unit.

The British Burns Association (BBA) by way of the National Burn Care Review has proposed guidance on recognizing a potentially complex burn injury that may require assessment and management at a specialist burns unit.^1^ They recommend that potentially complex burn injuries are discussed with the burns unit that can then advise on further management. These guidelines are not rigid but are designed to provide advice to those providing the initial treatment to patients with burn injuries.

There have also been similar guidelines recently published by a European working party for the management of partial thickness burns in a general hospital setting.^2^ These guidelines are aimed at nonspecialists and give advice on assessment and wound care as well as referral criteria for minor burns.

This study aimed to audit referral patterns of patients with minor burns in a British emergency department (ED) to see how closely the guidelines were being followed and whether there are particular groups of patients who are being either over- or underreferred to burns units.

## METHODS

We carried out a retrospective case note audit of all patients presenting to a single ED with a triage diagnosis of “burn.” The University Hospital Birmingham ED has 79,000 new attendances each year and the hospital is also the site of the West Midlands Regional Burns Unit. Tertiary referrals to the burns unit from other hospitals were excluded as these were felt to reflect a different clinical population.

**Figure 1:**
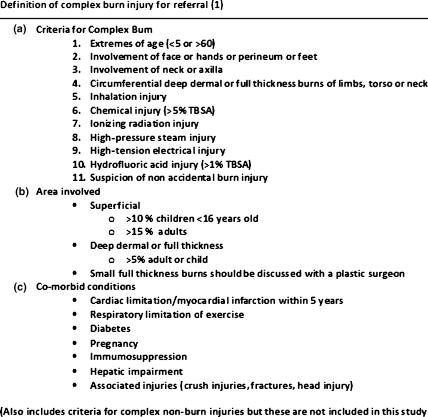
Definition of complex burn injury for referral.

The ED case notes were reviewed to gather data including details of the burn injury, examination findings, whether referral to another specialty was made, and outcome of the patient after leaving the department. This information was then compared with the referral guidelines produced by the BBA (Fig 1), allowing us to see how closely the guidelines were followed and which type of patients or injuries were managed appropriately.

For each patient the information available was used to determine whether the burn was complex in nature, whether a referral to the burns unit for advice or admission was warranted, and whether such a referral was made.

In many cases there was insufficient information in the case notes to decide whether a decision was appropriate or not. For example, if there was no TBSA or anatomical site recorded in the case notes then it was not possible to decide whether this could be classified as a complex burn that required referral. Conversely if there was any one of the referral criteria then it was deemed that a referral should have been made, even in the absence of other details.

The BBA guidance recognizes the fact that not all the referral criteria are absolute and some may be open to interpretation in the absence of other risk factors for a complex injury. For example, it states that some small injuries to the face or hands could be managed locally providing the patient is followed up in an ED clinic or similar. For the purposes of this study all patients who met any of the referral criteria were deemed to require a referral.

The regional burns unit is located on the same site as the ED in this study. Any patient referred to the burns service is seen in the ED by a member of the burns team who then decides on further management including whether admission of the patient is required or not. For the purposes of this study, any patient who was discussed with, or seen by, a member of the burns team before they left the ED was considered to have been referred.

Each patient was allocated into one of the following groups: “Appropriately referred” if they met referral criteria and were referred to the burns unit for either advice, outpatient follow-up or admission; “Appropriately not referred” if they had a noncomplex burn and were either discharged or followed up in the ED clinic; “Underreferred” if they met referral criteria but were not referred to the burns unit; “Overreferred” if they were referred to the burns unit despite not meeting the criteria for a complex burn injury; or “Not known” if there was insufficient information in the case notes to decide whether the management was appropriate or not.

## RESULTS

A total of 785 patients presented to the ED with a triage diagnosis of “burn” within the 1-year study period. Tertiary referrals from other hospitals were excluded, as were those patients whose notes were missing or did not include information about their outcome from the department, leaving 561 patients in this study. Details of the epidemiology of these patients have been presented previously.^3^

**Figure 2:**
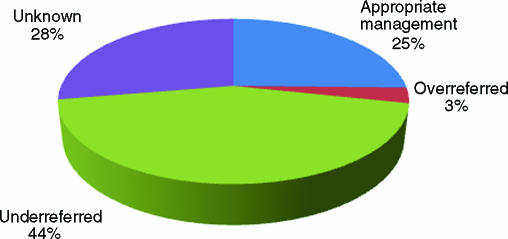
Patient outcome.

Overall, 142 patients (25%) were deemed to have had appropriate management according to the information available. This included 131 patients who required referral and were correctly referred, and further 11 who had noncomplex burn injuries and were correctly managed by the ED or general practitioner. A total of 247 patients were felt to have been underreferred in that they met referral criteria but were not referred to the burns service for an opinion, assessment, or admission. Of these patients, 107 patients were referred to other specialties or followed up in the ED clinic whereas the remaining 140 patients were discharged to the care of their general practitioner. Seventeen patients were overreferred to the burns unit despite not meeting referral criteria and the remaining 155 patients had insufficient information in the case notes. These results are summarized in Figure 2.

Of the 561 patients, 378 (67%) met at least 1 criterion for referral to the burns unit. However, of these 378, only 131 (35%) were actually referred. This means that of this sample there were potentially a further 247 patients who warranted referral to the burns unit if the referral criteria are being followed absolutely. In addition, it is likely that there were more patients who would have met the referral criteria if they had adequate information documented in the case notes. Therefore, even allowing for some flexibility in interpretation of the referral guidelines, it is clear that there are a significant number of patients presenting to this ED who may warrant referral because of complex burn injuries and are not currently being referred. The BBA guidelines highlight the need to discuss any case where there is doubt about the need for referral with the burns unit at the time of presentation.

**Figure 3:**
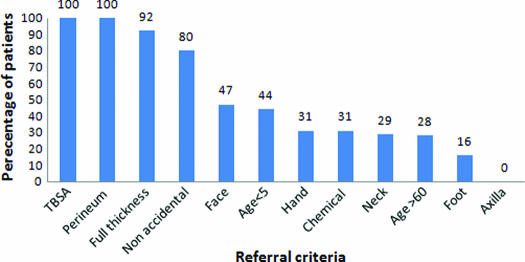
Proportion of patients correctly referred, by referral criteria.

Each referral criterion was studied individually to see whether there were particular groups of patients who were being managed inappropriately because of lack of familiarity with the guidelines. This sample of patients contained only a small number (7) of patients who required referral on the basis of TBSA alone but all these patients were correctly referred. Almost all (92%) of patients with full-thickness burns were also correctly referred, even in cases of small or mixed depth burns. However, other criteria were less well adhered to, including only 44% of children younger than 5 being referred and 31% of patients with burns to the hand. Figure 3 illustrates the proportion of patients who met each criterion who were appropriately referred.

Some patients met more than one of the referral criteria, for example a 2-year-old child with a hand burn. The number of referral criteria for each patient ranged from 0 to 4. Of the 247 patients who were underreferred, 189 (77%) met one referral criterion, 52 (21%) of them met 2 referral criteria, and 6 patients (2%) met 3 separate referral criteria and were not referred. It is likely that many of these patients who are not referred to the burns unit are being managed entirely appropriately by the experienced clinical staff in the ED. However, while there is room for some flexibility of the referral guidelines, it is more likely that any patient who meets 2 or 3 different referral criteria should be referred to the burns unit for specialist management and a failure to do this highlights a lack of familiarity with referral guidelines by the ED staff.

## DISCUSSION

Burn injuries are relatively common and account for 175,000 ED attendances in the United Kingdom each year^1^ and 1% of the case load of this department.^3^ Most of these burns are relatively minor and do not require formal fluid resuscitation but may require referral to a specialist burns unit to achieve the best outcome for the patient.

Burns can have many long-term sequelae including hypertrophic scarring, impaired function, and psychological distress. There is increasing recognition that these problems can be seen even after small burns—the so-called “small burn, big problem” patient.^4^

It has been shown that patients with small burns of less than 1% TBSA can suffer clinically significant levels of psychological morbidity several months after the initial injury.^5^ Problems with appearance and scars were also reported frequently in patients with minor burns^6^^,^^7^ and even small burns can have a significant impact on function, particularly when areas such as the hands are affected.

It has been highlighted in several previous papers that assessment of burns by nonspecialists is difficult and can be inaccurate.^8^ It has also been reported that in many cases of minor burns, the assessing doctors in the ED do not document enough information to enable them to make an accurate decision about the further management of the patient.^3^^,^^9^

Because of the potentially significant and long-term impact of burns, it is very important that all burn injuries are assessed, recognized, and referred correctly by the ED staff that are treating them. This may include nurse practitioners with extended roles as well as medical staff. Bezuhly and colleagues discuss the management of minor burns in EDs and call for stronger educational and clinical ties to be established between EDs and burns units.^9^ They suggest that surgeons and nurse specialists from the burns unit should increase their role as educators and provide information on treatment regimes and referral policies. We recognize the problems caused by rapid turnover of junior medical staff in EDs and suggest that assessment and management of burn injuries need to be covered more extensively in the curricula of both undergraduate medical degrees and the foundation program. Courses such as the Emergency Management of Severe Burns can teach specialist and nonspecialist staff a systematic and multidisciplinary approach to the patient with burns and should be considered by those working in EDs.^10^

A British Regional Burns Centre has previously reported a change in the pattern of adult burn referrals over the last 20 years. The Pinderfields Burns Centre has found that over the last 2 decades they have noted a significant increase in the number of patients admitted but found that there has been a decrease in the size and depth of burns as well as a decrease in the length of inpatient stay and mortality rate. The authors of this article feel that the increase in numbers is due to referring units strictly following the BBA referral guidelines, rather than an actual increase in the number of burn injuries in the region.^11^

In this study, any patient who met a single referral criterion and was not referred was considered to have been inappropriately managed in terms of referral outcome. It is likely that the majority of these patients had their burn injuries well managed with no long-term complications as the referral guidelines simply act to highlight those patients who are at risk of having a complex injury rather than those who will necessarily require specialist input. It is also likely that within the group of patients with insufficient information in their medical records, the majority will have been treated well and appropriately. Although the results of this study may seem to exaggerate the problem, the aim was to highlight current referral patterns along with implications for education, funding, and burns service organization.

We feel that the referral patterns demonstrated in this study are likely to be similar across the country. The ED in this study has the regional burns unit on site and it is, therefore, very simple for a member of the burns team to assess the patient in the department prior to their discharge and make a decision regarding their further management. It may be that the proportion of patients referred from other institutions without burns units are even lower because of the increased difficulty in transferring patients to another hospital for assessment. Medical staff in this department work entirely independently of the burns unit staff and are likely to have similar knowledge of burns as staff in other departments within the region or nationally.

This highlights the impact that adherence to these referral criteria could have on burns services in this country. It is likely that units will continue to see greater numbers of smaller, but potentially complex, burns. These are associated with shorter inpatient stays but also with increased number of outpatient attendances and clinic sessions. An increase in the number of patients referred with hand burns may require an increase in the number of dedicated hand therapists to optimize patient outcome. The result is likely to have more burns units dealing with larger numbers of small burns, many as outpatients, with fewer units dealing with larger and more complex burns. This coincides with the recommendations of the National Burns Care Review to restructure burn care services in the United Kingdom in line with differing levels of injury complexity.

## CONCLUSION

This study has found that overall only one quarter of the patients presenting to this ED are referred appropriately according to information recorded in the medical records and the referral guidelines produced by the BBA. This may be partly due to lack of familiarity with the guidelines, as well as the ability to manage some minor burns in the department with the knowledge that the regional burns unit is on site in case of later complications. Full-thickness and larger burns are consistently referred appropriately but burns of important anatomical sites and those in patients at the extremes of age are not always managed correctly and this can have long-term consequences for the patient who is potentially preventable.

The findings from this study are likely to be reflected nationally. We highlight the need for accurate assessment of minor burns, along with increased awareness of burns referral criteria, education of ED staff, and closer links between the burns unit and ED to obtain the best possible outcome for these patients.

Increased adherence to the national referral guidelines for burn injuries is likely to improve patient outcome at the expense of increased patient numbers and workloads in regional burns units that have implications for funding and service provision nationally.
